# Wideband tilted beam end-fire antenna using double semi-circular rings

**DOI:** 10.1038/s41598-026-35414-8

**Published:** 2026-01-17

**Authors:** Amitkumar Patel, Chinthana Panagamuwa, William Whittow

**Affiliations:** Wolfson School of Mechanical, Electrical and Manufacturing Engineering, Loughborough University, Loughborough, LE11 3TU UK

**Keywords:** Engineering, Physics

## Abstract

This article presents a novel wideband (WB) tilted-beam end-fire planar antenna for microwave and millimeter-wave (mmWave) applications. The antenna comprises of a microstrip fed double semi-circular rings over a curvilinear slotted ground plane. It is impedance matched over a broad frequency range from 11.5 to 62.5 GHz, covering the 5G New Radio (NR) mmWave bands n257, n258, n260, n261, and partly covering the unlicensed 60 GHz band. Across this entire band, the antenna exhibits a return loss better than 12 dB and a gain exceeding 6.5 dBi, with a peak gain of 11.6 dBi at 40 GHz. The overall electrical size of the antenna is 1.28 $\times$ 1 $\times$ 0.08 $\lambda _0^3$, where $\lambda _{0}$ corresponds to the free-space wavelength at 32 GHz. Within the 24–40 GHz frequency range, corresponding to a 50% fractional bandwidth and covering the four 5G NR bands, end-fire radiation is achieved with a tilted beam angle of $\textrm{65}^{\circ }$ ± $\textrm{10}^{\circ }$. A prototype of the antenna is fabricated and experimentally characterized. The measured results show good agreement with full-wave simulations, validating the proposed design. Owing to its compact planar geometry, wide bandwidth, and high gain, the antenna is a strong candidate for future high-data-rate wireless communication systems.

## Introduction

The rapid advancement of modern wireless communication systems necessitates broad bandwidth and high data rates to meet the demands of a growing number of users^1,2^. While the Sub-6 GHz frequency band has partially addressed these needs, it has not fully met the expectations for 5G^3^. Recognizing this, the International Telecommunication Union (ITU) has identified new 5G new radio (NR) frequency range 2 (FR2) frequency bands for future mmWave applications^4^.

According to regulations issued by the European Union and the Federal Communications Commission, several millimeter-wave (mmWave) frequency bands have been allocated for 5G networks. These include the n257 (26.5-29.5GHz), n258 (24.25–27.5 GHz), n260 (37–40 GHz) and n261 (27.5–28.35 GHz) bands. Additional allocations at 24 GHz (24.25–24.45 GHz and 24.75–25.25 GHz), 29 GHz (29.1–29.25 GHz), 32 GHz (31.4–33.8 GHz) are often used for fixed-links, and the unlicensed 60 GHz band (57–63.5 GHz) for WiGig^4,5^.

Modern wireless communications demand a simple and compact geometry, particularly for planar antennas, to facilitate easy mounting and integration with other physical modules^6,7^. Furthermore, a small geometry antenna should provide a suitable gain-bandwidth product. Many different types of broadside and end-fire antennas have been proposed for wideband applications based on microstrip and substrate integrated waveguide (SIW) techniques^3,5,6,8–10^.

End-fire antennas based on microstrip patches, such as helical antennas and Yagi antennas, are widely used due to their consistent radiation patterns. While these antennas give high gain, they often lack sufficient bandwidth^11,12^. A popular category of antennas is based on the SIW technique, known for its planar geometry and high gain^13^. However, fabricating vias inside the structure, specifically for mmWave frequency bands, using standard printed circuit board technology poses challenges and limits the bandwidth. To address these issues, spoof surface plasmon polaritons (SSPPs) based end-fire antennas have been proposed^14–16^. These antennas employ symmetric strip elements to support both odd and even resonant modes, while asymmetric patches and loading deflectors are introduced to steer the radiation beam in the quasi-E plane^12^. However, the main beam deviated from the desired angle and generated higher side-lobe levels (SLLs).

**Fig. 1:**
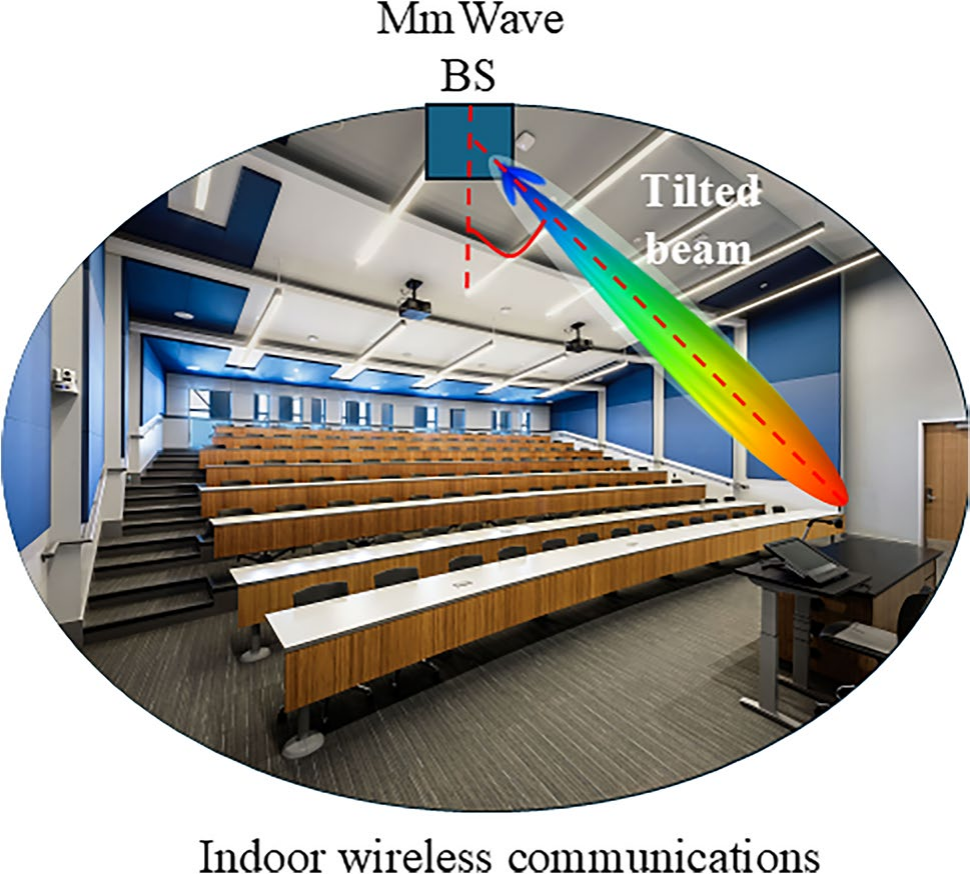
Potential application of a tilted beam antenna to provide 5G NR connectivity to the podium area of a lecture theater.

Tilted beam antennas play a crucial role in 5G mmWave base station (BS), serving as key technologies to enhance transmission quality, channel capacity, and reduce co-channel interference, particularly in densely populated urban areas where BS are concentrated^5,17^. Moreover, tilted beam BS provides flexibility in coverage direction which is useful in various indoor diverse applications. Figure 1 shows the potential application of the tilted beam antennas in future 5G mmWave indoor wireless communications. This antenna helps in managing the coverage radius and improve the signal-to-noise ratio. Beam tilting can be achieved through mechanical and electronic means^18–22^. Mechanically steerable antennas give better gain but require large volumes for phase shifters^19^. Conversely, electronically controlled beam tilting typically uses parasitic varactor diodes, PIN diodes, micro-electro-mechanical-switches (MEMS), and other phase-shifting devices, which are compact and easy to configure^17,23^.

Many antennas proposed in the literature encounter challenges in achieving the radiated beam in the tilting direction^13,19,23^. Some struggle with narrow operating bandwidths, limited gain^24^, and requires active elements or parasitic (metamaterials-based geometries) components to adjust the tilting angle, leading to increase antenna size^20^.

Many reported works have proposed to improve the bandwidth and stabilize the beam direction across an operating band^25–27^. These antennas radiate a beam in the end-fire direction. This SSPP types of modification in the design improved the bandwidth as well as reduced the SLL significantly^27^. However, such geometrical adjustments have typically led to an increase in the overall size of the antenna structure.

Overall, most of the designs used parasitic elements or MTMs to tilt the main beam direction , which reduced the bandwidth and limited gain. To address these limitations, a novel tilted beam end-fire antenna based on double semi-circular rings has been proposed in this article. The incorporation of a 2D grooved structure has been employed to enhance gain and stabilize the radiation pattern. Following are the key features of the proposed tilted beam end-fire antenna:

1. Notably, this antenna boasts a compact and straightforward geometry, offering a wide bandwidth spanning from 11.5 to 62.5 GHz (132.1% FBW), and 24 to 48 GHz (75% FBW) for stabilized radiation patterns.(Note: due to limited measurement facility at host university we have carried out radiation pattern measurement up to 40 GHz)
2. It achieves a gain of more than 6.5 dBi across the entire operating band, with a tilted beam radiation pattern at $\textrm{65}^\circ$ ± $\textrm{10}^\circ$.
3. Experimental results corroborate the simulated outcomes, validating the efficacy of the design.

The organization of the paper is as follows: “Design and analysis of Double semi-circular rings as tilted beam end-fire antenna” describes detailed design theory, evaluation of the antenna, the parametric variation and impact on the performance; The fabrication of the antenna, and experimental results are shown in “Fabrication and Experimental Results”; A comparison with the previously reported works is shown in “Comparison and Discussion” and finally, some important conclusions on the proposed design are given in “Conclusion”.

## Design and analysis of double semi-circular rings as tilted beam end-fire antenna

**Fig. 2:**
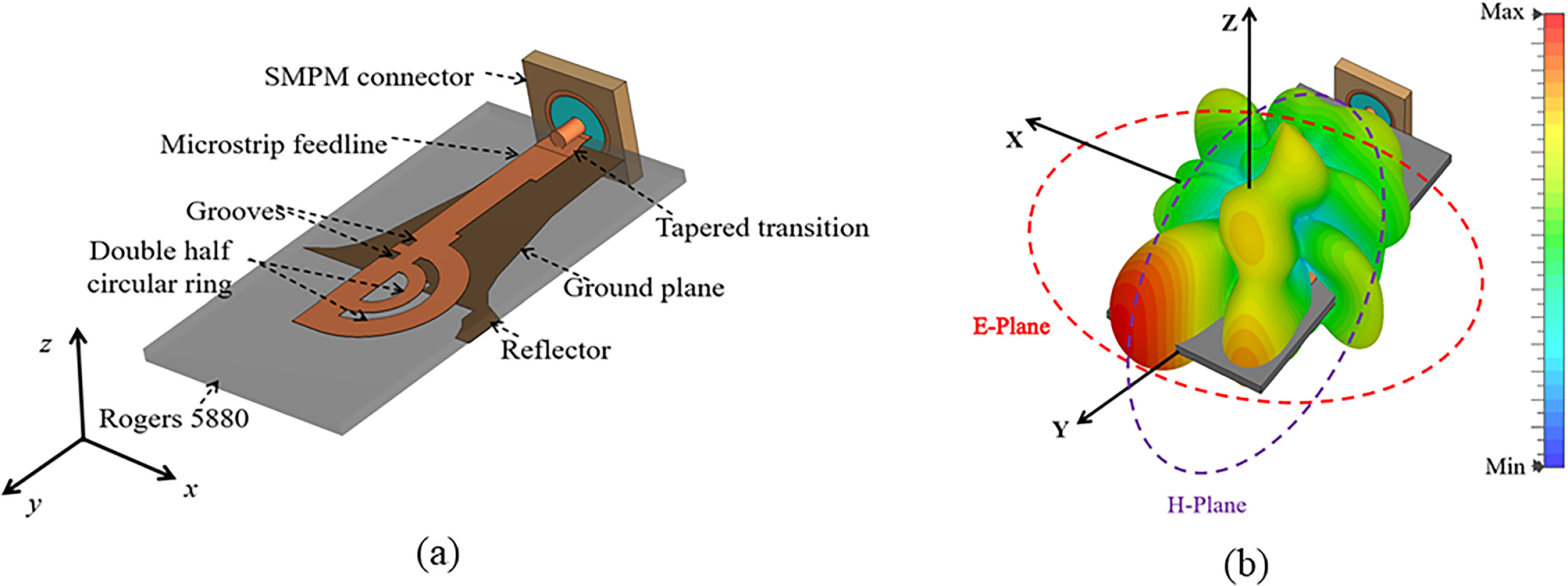
(**a**) Geometry of the proposed tilted beam end-fire antenna using double semi-circular rings with SMPM connector; and (**b**) Orientation of the E- and H-planes and the simulated 3D gain plot of the proposed antenna at 40 GHz.

The geometry of the proposed double semi-circular rings as tilted beam end-fire antenna is shown in Fig. 2a. The structure compromises double semi-circular ring fed with microstrip line. A tapered transition has been engineered to facilitate the connection between the feedline and sub-miniature push-on micro (SMPM) connector, capable of operating up to 65 GHz.

The optimization of the double semi-circular rings and partial ground plane ensures that the proposed design radiates a tilted beam in the end-fire direction across a wide frequency range. Figure 2b shows the orientation of the E- and H-planes along with the simulated 3D gain pattern of the proposed antenna at 40 GHz. To maintain the beam radiation consistently within the operating band, two rectangular grooves are introduced in the feed line, controlling the direction of current flow. Especially, no additional parasitic elements or metamaterials are integrated into the proposed geometry to tilt the beam direction. This simplicity makes the proposed design highly suitable for integration into cellular networks.

### Modal field distributions of a circular ring antenna

**Fig. 3:**
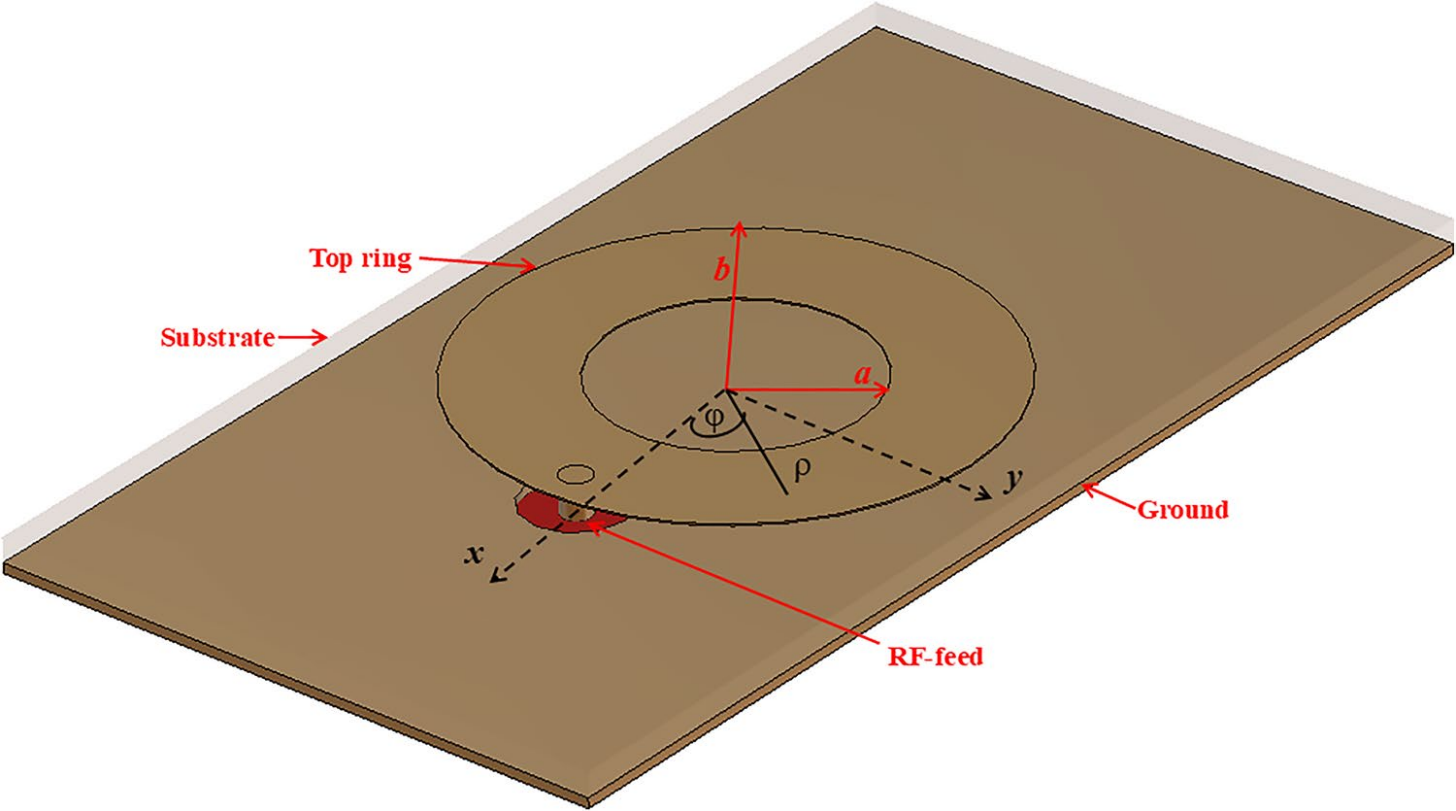
Geometry of a circular ring microstrip antenna.

    To understand the radiation field of the microstrip circular ring, an analytic modal field analysis has been carried-out in^28^. A circular ring antenna consists of a radiating ring-shaped patch, typically printed on a dielectric substrate with a ground plane on the opposite side, as shown in Fig. 3. In effect, it is an intermediate geometry between a printed loop and a patch.

The geometry of circular ring is substantially smaller than that of the corresponding patch antenna. The mean ring’s circumference is equal to the guide wavelength of the microstrip patch. Depending on the ring width, the antenna is operated in either $\textrm{TM}_{11}$ or $\textrm{TM}_{01}$ modes^28^. The ring antenna operated in the $\textrm{TM}_{11}$ mode offers a high input impedance and narrow bandwidth. The inner and outer radii of the ring define its resonant behavior, and separation of resonant modes. Moreover, its performance can be tailored by adjusting geometric parameters, feed configuration, and substrate properties. When this antenna is operated in the $\textrm{TM}_{21}$ mode, it provides several times higher bandwidth compared to $\textrm{TM}_{11}$ mode but requires a larger size. However, it has been shown that this antenna is a good resonator for $\textrm{TM}_{1m}$ modes (m odd) and a good radiator for $\textrm{TM}_{1m}$ modes (m even)^28^.

As shown in Figure 3, *a* and *b* are inner and outer the radii of the circular ring. When the substrate thickness of the antenna is very small compared to the operating wavelength and outer peripherals act as magnetic walls, the circular cavity model can be used to study the operating modes of the ring antenna.

The general wave solution for this circular cavity model is given as^28^.

1 $$\begin{aligned} E_z = E_0 \left[ J_n(k\rho ) Y_n'(ka) - J_n'(ka) Y_n(k\rho ) \right] \cos (n\phi ) \end{aligned}$$

where $J_n(.)$ and $Y_{n}(.)$ are the Bessel functions of the first and second kind, and of order n, respectively. The surface current on the inner edge of the metal ring is given by

2 $$\begin{aligned} J_\phi = \frac{j n E_0}{\omega \mu \rho } \left[ J_n(k\rho ) Y_n'(ka) - J_n'(ka) Y_n(k\rho ) \right] \sin (n\phi ) \end{aligned}$$

3 $$\begin{aligned} J_\rho = -\frac{j k E_0}{\omega \mu } \left[ J_n'(k\rho ) Y_n'(ka) - J_n'(ka) Y_n'(k\rho ) \right] \cos (n\phi ) \end{aligned}$$

As per the boundary conditions applied at $\rho$=*a* and $\rho$=*b*; the radial component of the surface current must vanish along the edges.

4 $$\begin{aligned} J_\rho (\rho = b) = H_\phi (\rho = b) = 0 \end{aligned}$$

This leads to the well-known characteristic equation for the resonant modes:

5 $$\begin{aligned} J_n'(k b)\, Y_n'(k a) - J_n'(k a)\, Y_n'(k b) = 0 \end{aligned}$$

The various roots of equation (5) can be obtained based on the various values of *a*, *b*, $\epsilon _{r}$ and *n*. Let us consider these roots by $k_{nm}$ for the resonant modes $\textrm{TM}_{nm}$ and form $\chi _{nm}$ such that $\chi _{nm}$= $k_{nm}$ *a*. The integer n denotes the azimuth field variation as per $cosn\phi$, while the integer m represents the variation of fields across the width of the ring. The modes field pattern and current distributions for some of the modes on the microstrip ring are illustrated in^28^. It provides the design base for quasi-beam/tilted-beam radiating antenna.

The following observations have been made based on modal characteristics and radiation patterns illustrated in^29^ of the ring : the $\phi$-symmetric modes ($\textrm{TM}_{0m}$) possess nulls in the normal direction. $\textrm{TM}_{1m}$-modes are only radiate in the normal direction. When the ring width exceeds half the guide wavelength, higher-order $\textrm{TM}_{nm0}$-modes (n $\ge$ 0, m > 1) appear^29^. The resonance of the dominant mode is given by the lowest value of $k\rho$.

### Evolution of the double semi-circular rings antenna

The evolution and analysis of the double semi-circular rings antenna are crucial steps in understanding its design principles and performance characteristics. This section delves into the iterative process of refining the antenna geometry and conducting extensive simulations to optimize its parameters.

**Fig. 4:**
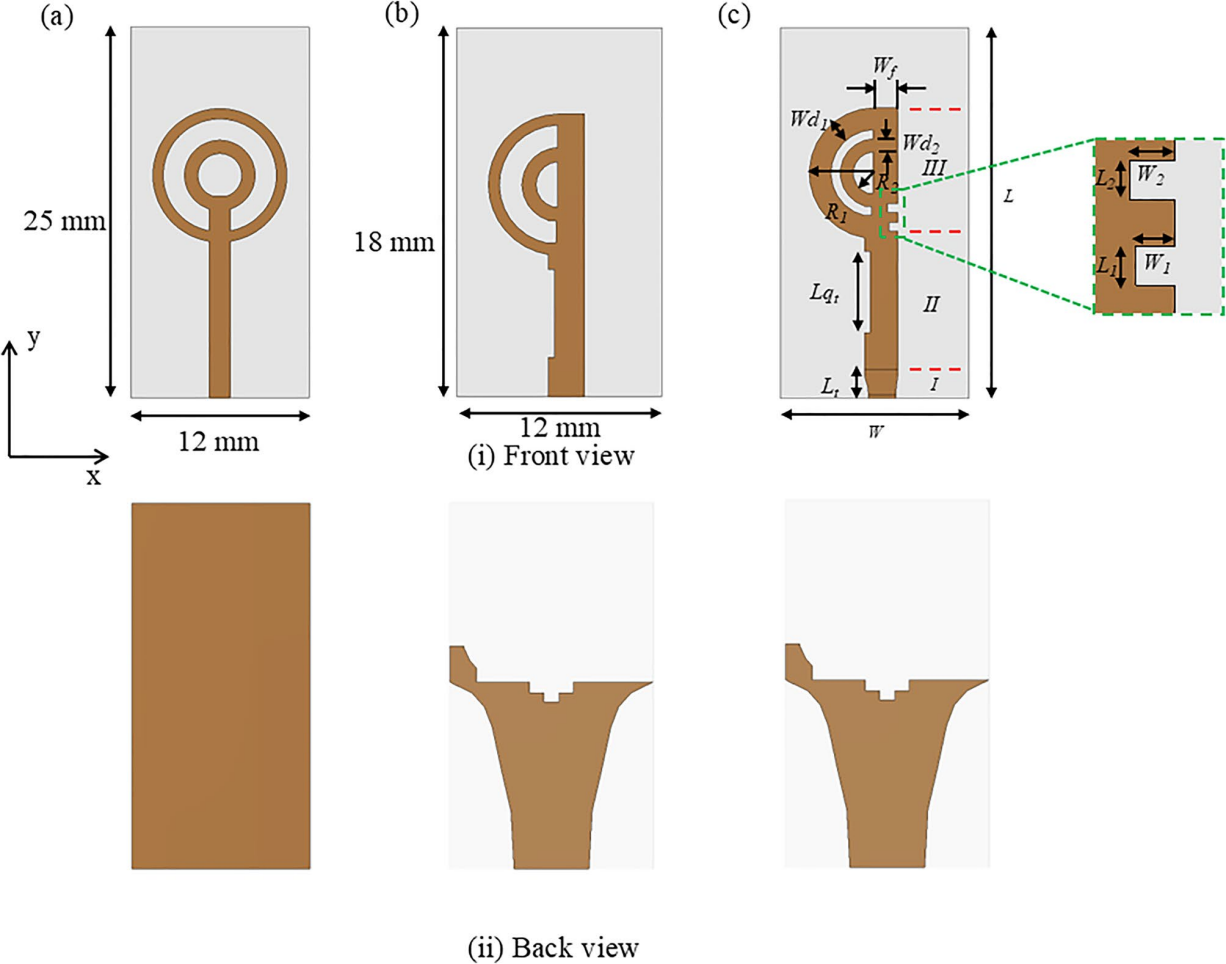
Evolution of the proposed tilted beam end-fire antenna (**a**) Double circular rings with full ground; (**b**) Double semi-circular rings with curvilinear ground plane, reflector; and slots and (**c**) Double semi-circular rings with two grooves and curvilinear ground plane, reflector, and slots.

**Fig. 5:**
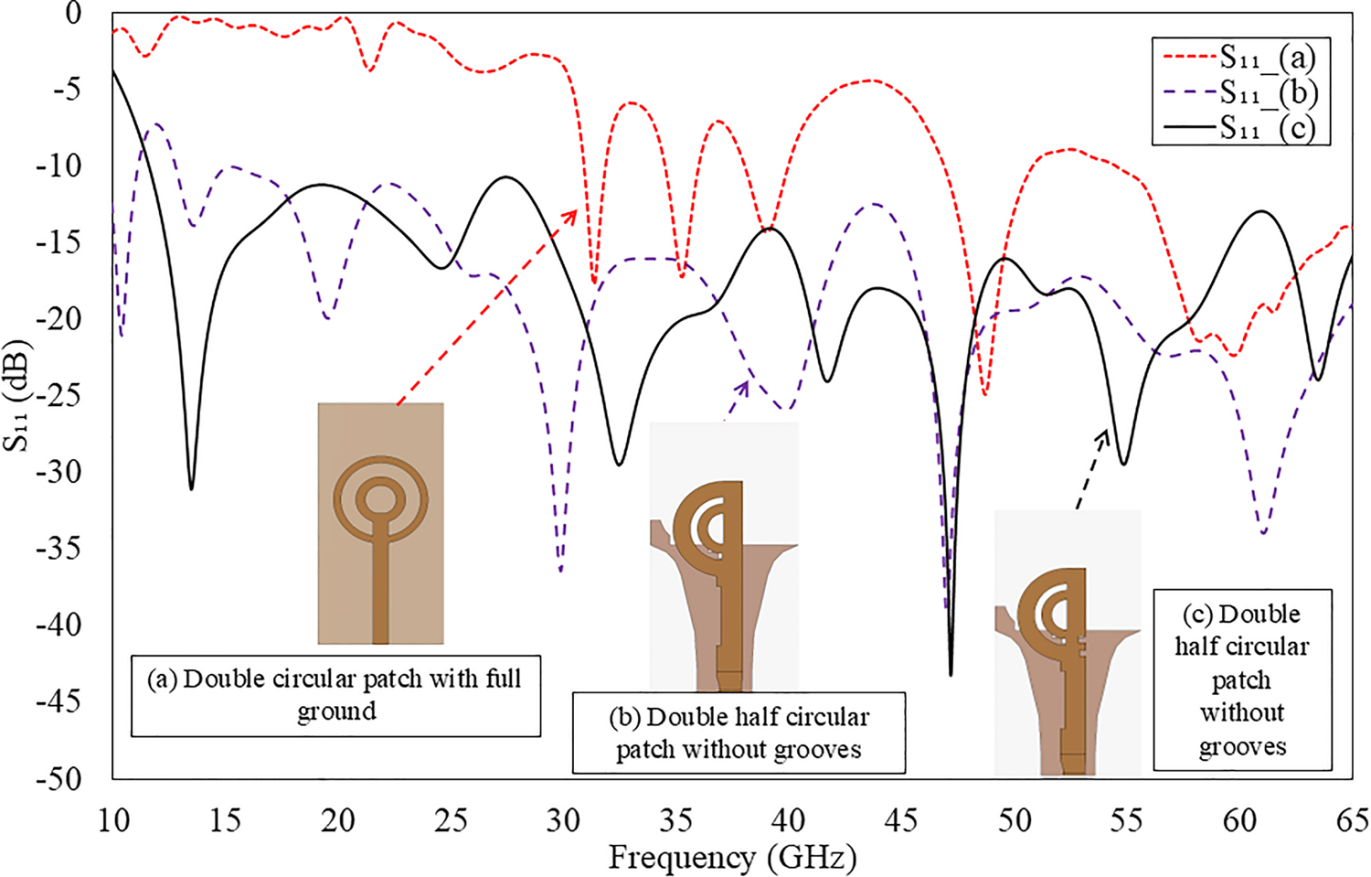
Simulated reflection coefficients of the proposed antenna for different evolution stages as shown in Fig. 4.

Figure 4 illustrates the evolution of the proposed double semi-circular rings patch as tilted beam end-fire antenna design. In Fig. 4a, a circular microstrip ring with a full ground plane functions akin to a monopole antenna, emitting a beam in the broadside direction when operating in its fundamental mode^28,29^. However, this configuration has limitations, including a narrow operating bandwidth and challenges in maintaining the beam direction in the end-fire orientation. Additionally, the antenna exhibits large SLLs due to poor impedance matching with free space.

The simulated reflection coefficient for the double annular circular rings with a full ground plane is depicted in Fig. 5. It demonstrates a -10 dB impedance bandwidth (IBW) spanning from 35 to 46.5 GHz.

**Table 1:** Dimensions of the optimized double semi-circular rings antenna.

Parameter	Dimension (mm)	Parameter	Dimension (mm)	Parameter	Dimension (mm)
*W*	12	$w_{d1}$	1.5	$L_1$	0.5
*L*	18	$w_{d2}$	0.9	$L_2$	0.6
$R_1$	4.5	$L_t$	1.7	$L_f$	7.5
$R_2$	2.37	$w_f$	1.7	$L_{g1}$	0.6
$R_c$	3	$W_g$	4.8	$w_{g1}$	1
$R_g$	4.2	$L_g$	10.94	$w_{g2}$	2.8
$W_1$	0.6	$W_2$	0.7	$L_{qt}$	5.6

To enhance the antenna’s bandwidth, gain performance, and maintain a consistent beam direction without enlarging the structure’s size, the author proposes a design featuring double semi-circular rings with a curvilinear slotted ground plane, illustrated in Fig. 4b. The overall dimensions of the antenna are shown in Table 1. This configuration achieves a -10 dB IBW spanning from 13 to 65 GHz, shown in Fig. 5. The design modification significantly improves the bandwidth and produces a single beam in the tilted quasi-E plane. However, the direction of the tilted beam varies from $\textrm{37}^{\circ }$ to $\textrm{75}^{\circ }$ in the azimuth plane as the operating frequency shifts from 18 to 52.5 GHz. For frequencies below 18 GHz and above 48 GHz, the main beam is primarily directed in the boresight direction. The proposed design supports different modes: lower frequency ranges from 18 to 30 GHz and higher frequency ranges from 30 to 48 GHz.

To ensure the radiation beam remains in the same direction, two optimised rectangular grooves are created in the feedline near the double circular rings, as shown in Fig. 4 (c). These grooves effectively control the flow of the current and steer the beam in a consistent direction without compromising the operating bandwidth. As a result, the antenna achieves a reflection coefficient better than -10 dB over a frequency range from 11.5 to 65 GHz that is shown in Fig. 5. Although the proposed design offers a wide IBW, it maintains the tilted beam in the azimuth plane from 24 to 48 GHz. Below 24 GHz and above 48 GHz, the beams are radiated in the boresight direction.

### Design of double semi-circular rings as tilted beam end-fire antenna

The doublesemi-circular rings based tilted beam end-fire antenna design is represented in Fig. 4c. It shows that the proposed antenna is created from simple two semi-circular rings. The radius of the outer ring can be obtained using Eq.(6)^28^.

6 $$\begin{aligned} R = \frac{\chi _{nm} c}{2\pi f_r \sqrt{2\epsilon _r}} \end{aligned}$$

where $f_r$ is the resonant frequency, $\epsilon _r$ is the permittivity of the substrate, and $\chi _{nm}$ is the $m^{th}$ zero of the derivative of $n^{th}$ order of the Bessel’s function. Hence, the radius of the ring is crucial for designing the flow of higher-order modes. The radii of circular rings are chosen as 4.9 mm (with a thickness of 0.7 mm) and 2.3 mm (with a thickness of 0.58 mm), respectively. They allow the flow of higher order modes. Additionally, a substrate with an $\epsilon _r$ > 1 can generate a beam with a narrow beamwidth^25^. The structure has been excited by a 50 $\Omega$ microstrip feed line with a width and length of 1.4 mm and 6 mm, respectively. The proposed antenna size measures 18 $\times$ 12 $\textrm{mm}^{2}$ and is constructed on an RT Duroid 5880 substrate from Rogers, with a thickness of 0.78 mm, a dielectric constant ($\epsilon _r$) of 2.2, and a loss tangent (tan $\delta$) of 0.0009 (measured at 10 GHz, as per the available datasheet from the manufacturer). Both the radiating patch and ground are copper, each with a thickness of 0.035 mm.

The physical parameters of both the circular rings and the curvature of the ground plane are pivotal elements in achieving the desired performance of the proposed antenna. Parametric analysis has been conducted on these geometrical parameters to assess their impact on reflection coefficients and beam direction. The simulated results have been obtained using commercially available Computer Simulation Technology (CST) Microwave Studio software.

**Fig. 6:**
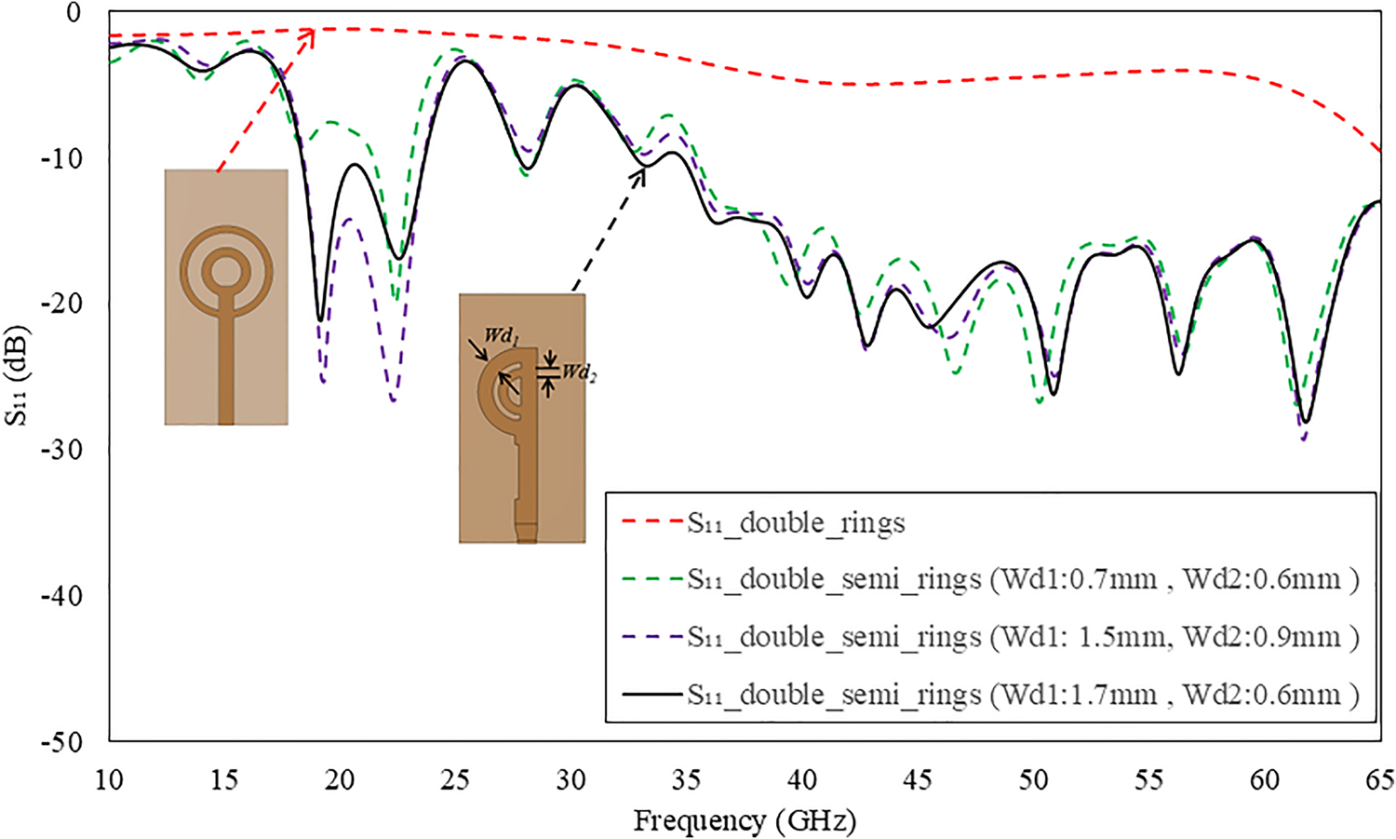
Comparison of simulated reflections coefficients for double full and semi-circular rings with a full ground plane.

**Fig. 7:**
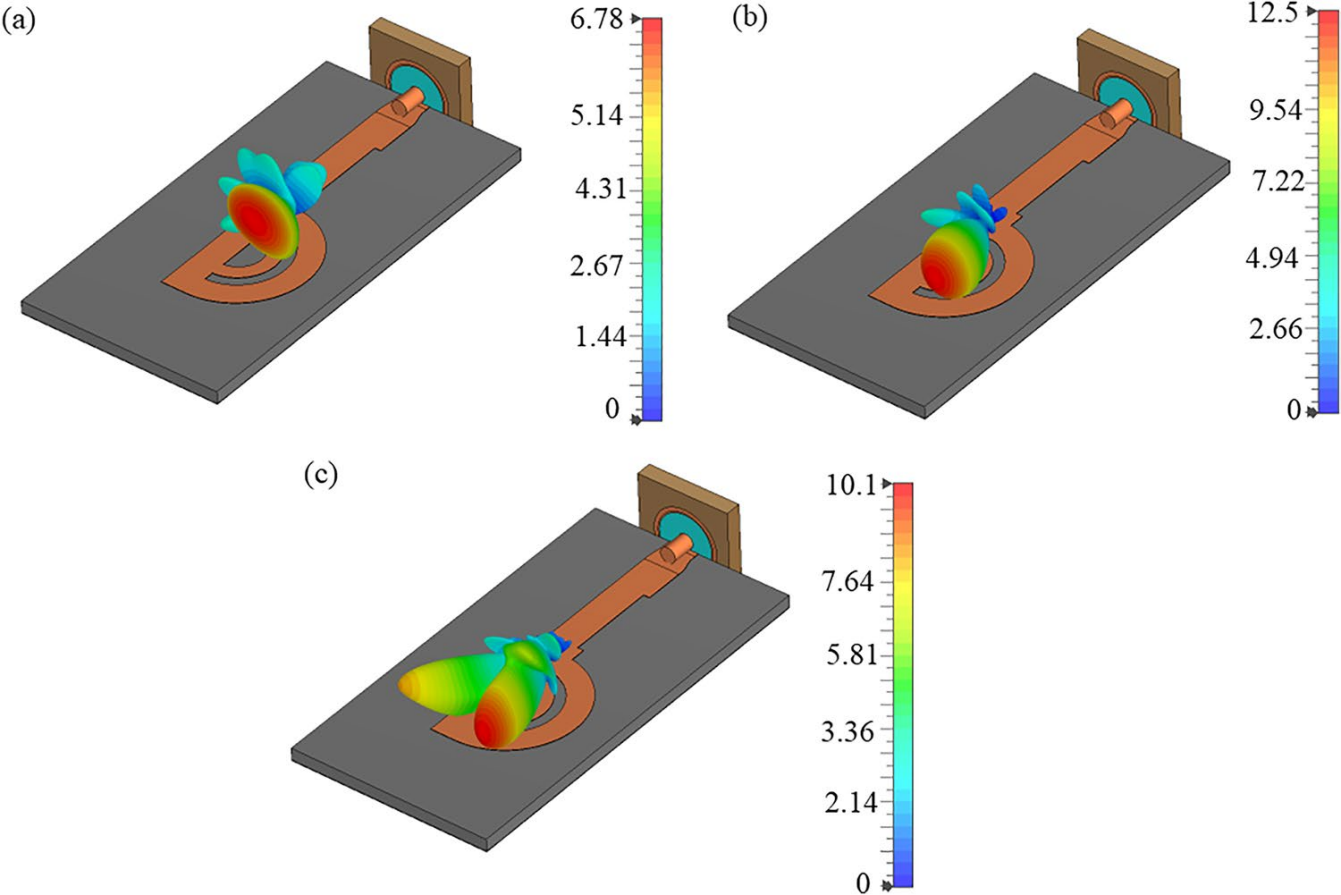
Simulated 3D linear realized gain plots for the proposed double semi-circular ring antenna at (**a**) 23 GHz; (**b**) 38 GHz; and (**c**) 46 GHz.

Initially, the double circular rings are transformed into semi-circular rings while retaining the full ground plane, as illustrated in Fig. 4. The thickness and diameter of both rings were optimized to attain a wide operating bandwidth. A comparison of the simulated reflection coefficients for double full and semi-circular rings is depicted in Fig. 6. It reveals that the semi-circular rings yield a -10 dB IBW ranging from 18.4 to 23.5 GHz, and 35 to 65 GHz. The simulated 3D linear gain plots at 23 GHz, 38 GHz, and 46 GHz are presented in Fig. 7. Note, the linear scale was used to allow better visualization of the radiation patterns. Although there is a significant improvement in bandwidth performance, the direction of beams at lower frequencies (23 GHz) tends towards $\theta$ = $\textrm{30}^{\circ }$ and then becomes dual-beam for frequencies > 46 GHz.

### Optimization of ground plane

**Fig. 8:**
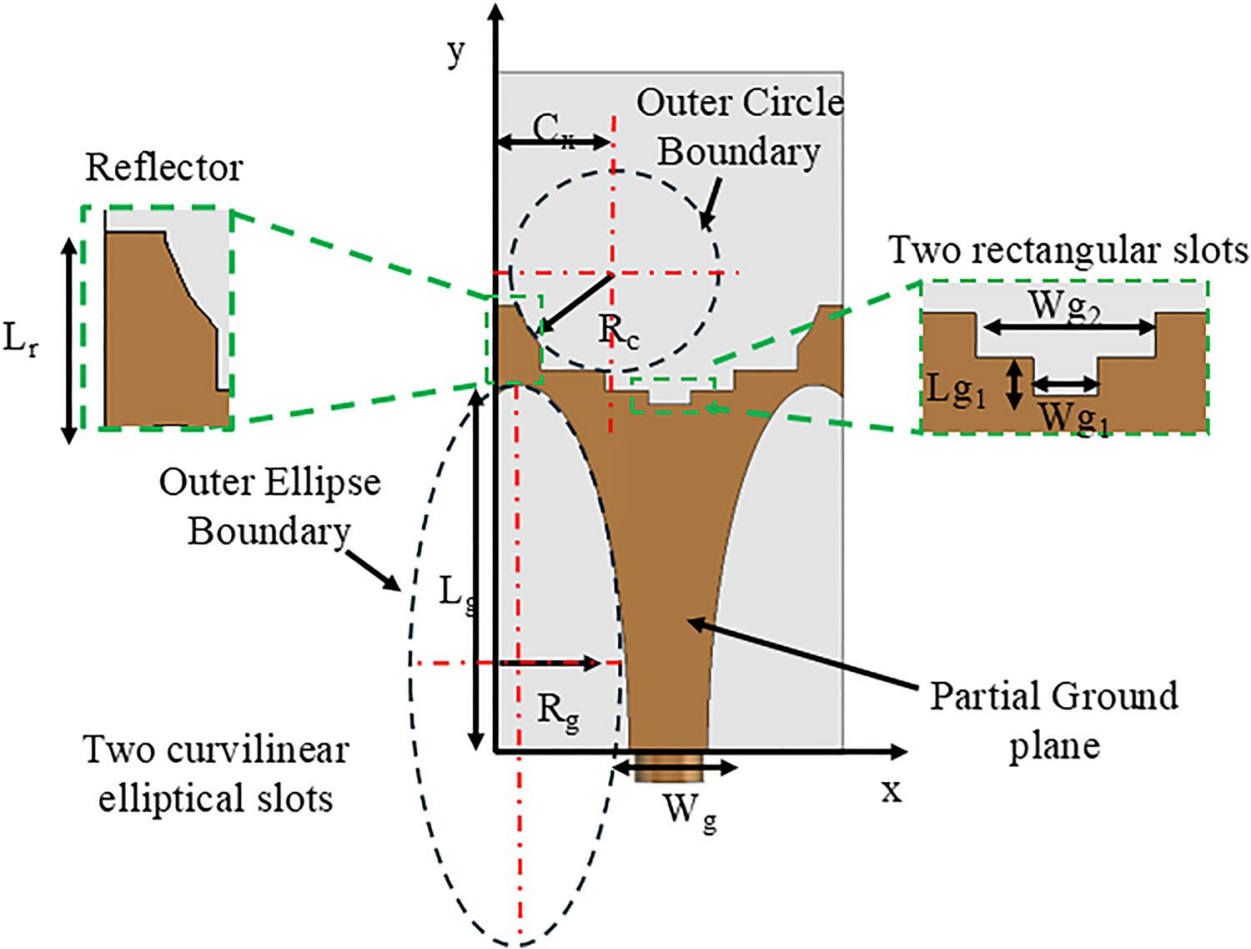
Optimized ground plane in the proposed antenna.

**Fig. 9:**
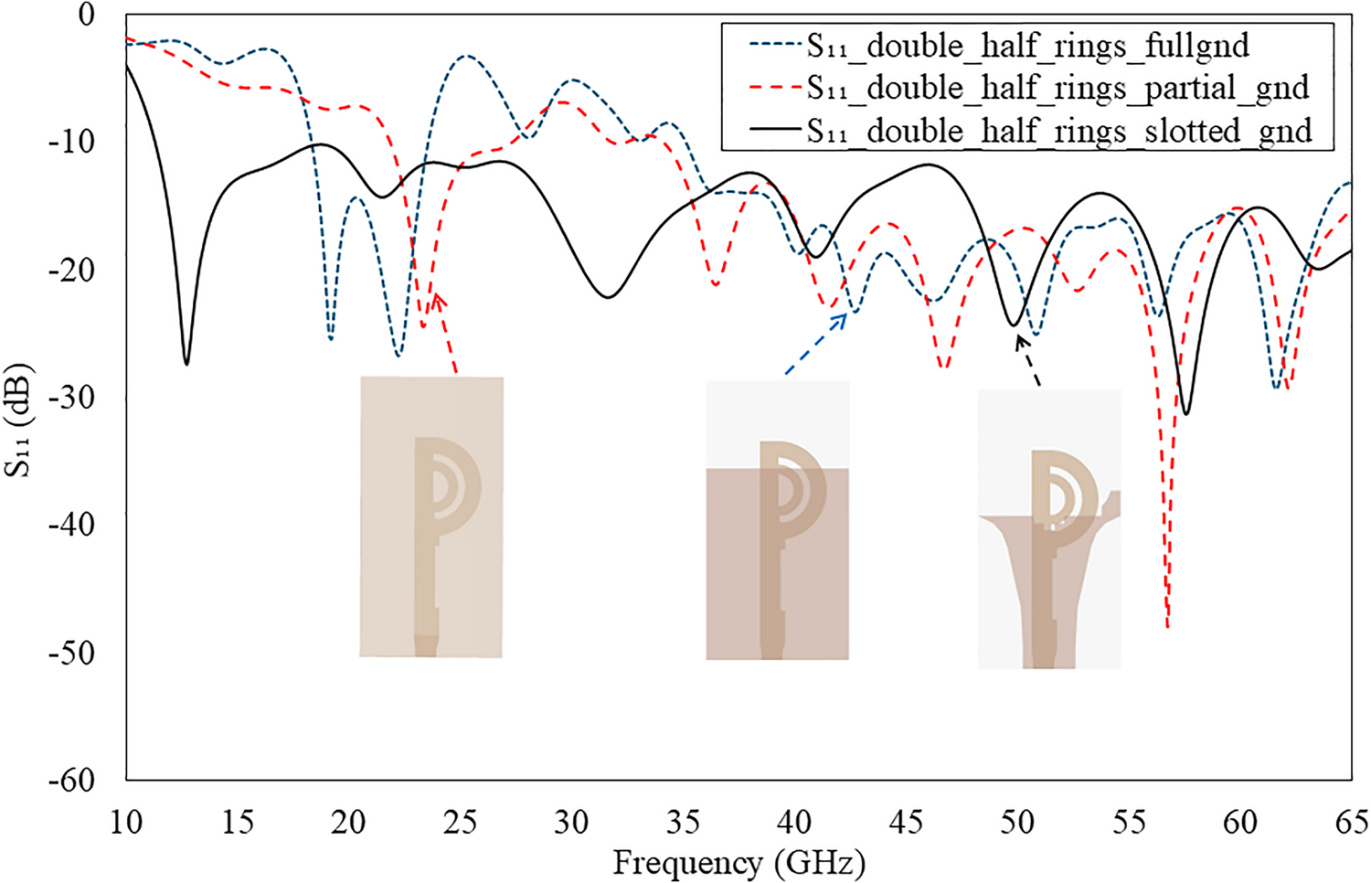
Comparison of simulated reflection coefficients of the double semi-circular rings with respect to variations in the ground plane.

To enhance the performance of the proposed double semi-circular rings antenna in terms of bandwidth, beam direction, and radiation patterns, modifications were made to the ground plane as depicted in Fig. 8. The stepwise modifications in the ground plane, evaluated in terms of reflection coefficients, are illustrated in Fig. 9. Compared to a full ground plane, both a partial ground plane without any slots (from 22 to 27.5 GHz, and 34 to 65 GHz) and a slotted partial ground plane with a reflector (from 11.3 to > 65 GHz) achieved a wide operating bandwidth.

### Stabilizing the tilted beam in the end-fire direction

**Fig. 10:**
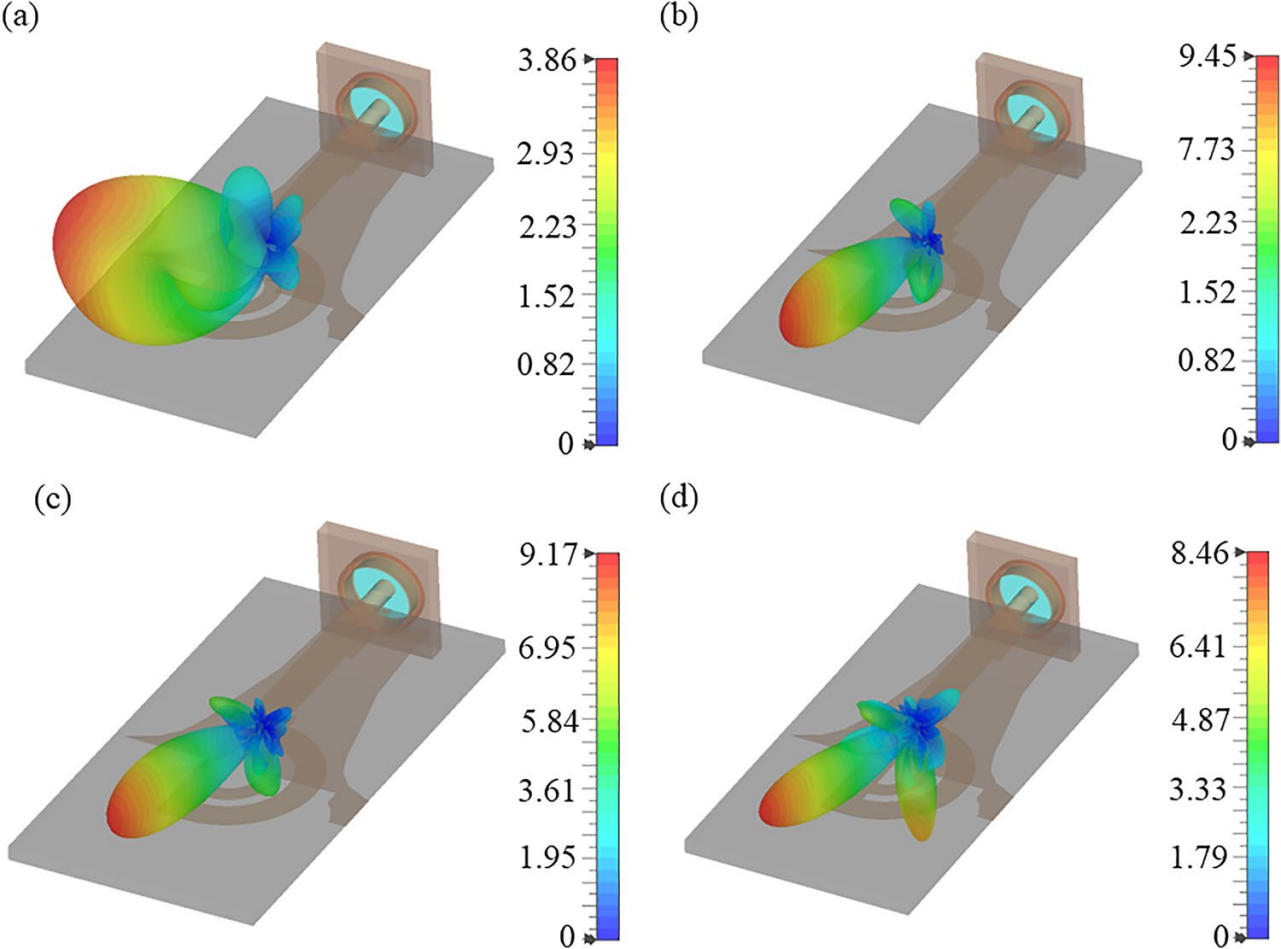
Simulated 3D linear realized gain plots of the double semi-circular rings with slotted ground plane and reflector at (**a**) 24 GHz; (**b**) 38 GHz; (**c**) 46 GHz; and (**d**) 52 GHz.

This section explores the mechanism of beam tilting and proposes a technique to stabilize the beam direction in the azimuth plane. The beam tilting approach relies on the generation of higher ordered modes in the microstrip circular ring^22^. To excite the higher order modes, the feedline is asymmetrically designed, resulting in a conversion from quasi-TEM to higher order modes. Current with different modes encounter two distinct impedance paths at the end of the feedline where the double semi-circular ring design is connected. As a result, the beam direction changes depending on the operating frequency, as depicted in Fig. 10.

**Fig. 11:**
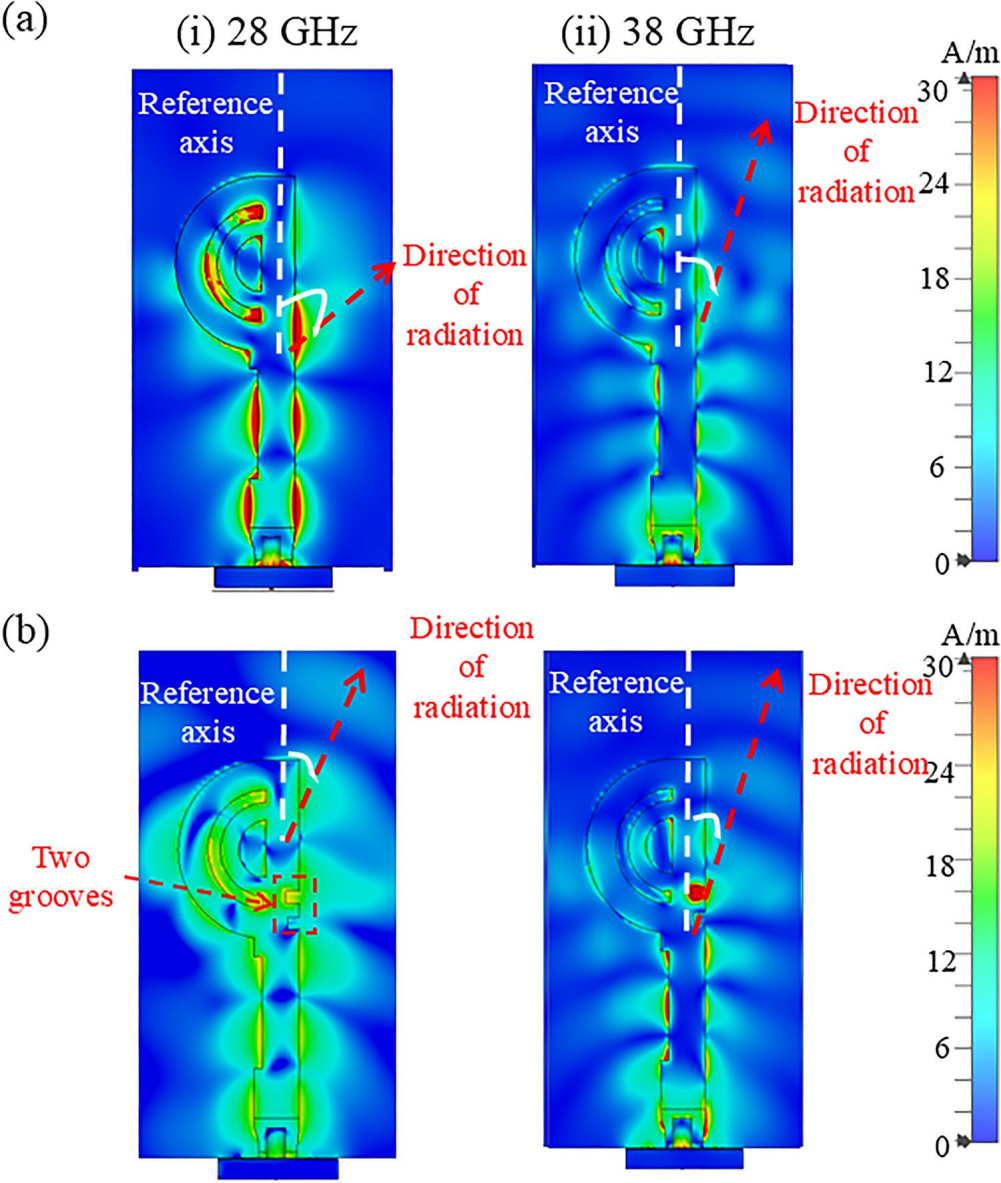
The surface current flows at 24 and 38 GHz (**a**) without; and (**b**) with corrugated slots in the feedline.

At lower frequency bands, most of the current approaches from the double semi-circular ring, while at higher frequencies, the current flows in a straight path with slight leakage towards double circular rings, as shown in Fig. 11a. Consequently, the direction of the radiation beam varies for these two distinct frequency bands. To equalize the direction of radiation beams in both operating bands, two rectangular groove slots, approximately quarter wavelength size, are designed in the feedline, as shown in Fig. 4c. These grooves behaves high impedance surface at and above resonant frequencies. The detailed analysis of 2D corrugated surface is reported in^30^ This design ensures that all the current flows from the feedline to the double semi-circular rings. As a result, the direction of the beams for all the operating frequencies becomes almost the same. The current flow at both frequency bands with the corrugated slots is depicted in Fig. 11b. The dimensions of the two grooves are optimised in such a way that they do not affect the IBW.

**Fig. 12:**
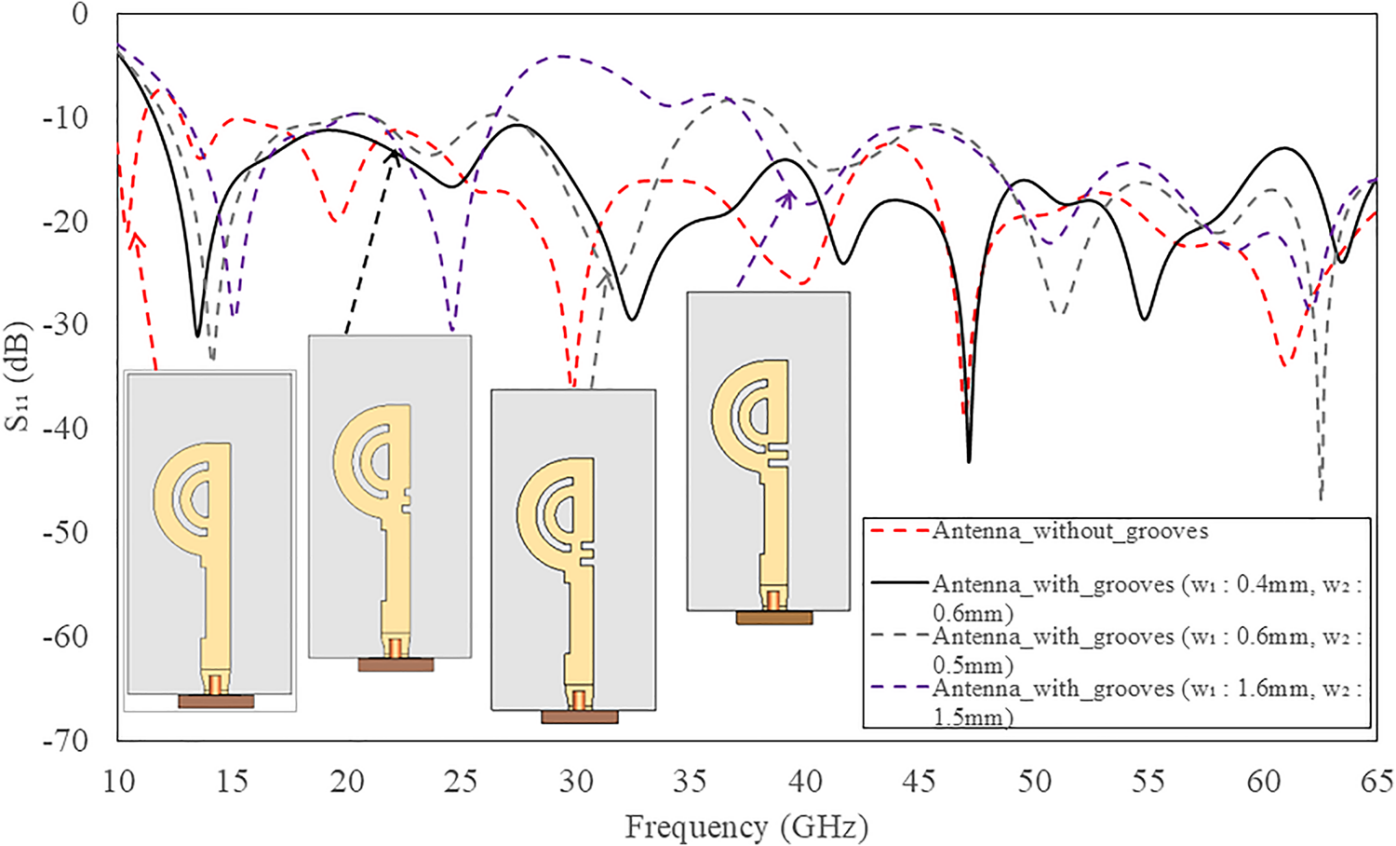
Comparison of simulated reflection coefficients of the proposed double semi-circular rings antenna with and without grooves in top patch.

**Fig. 13:**
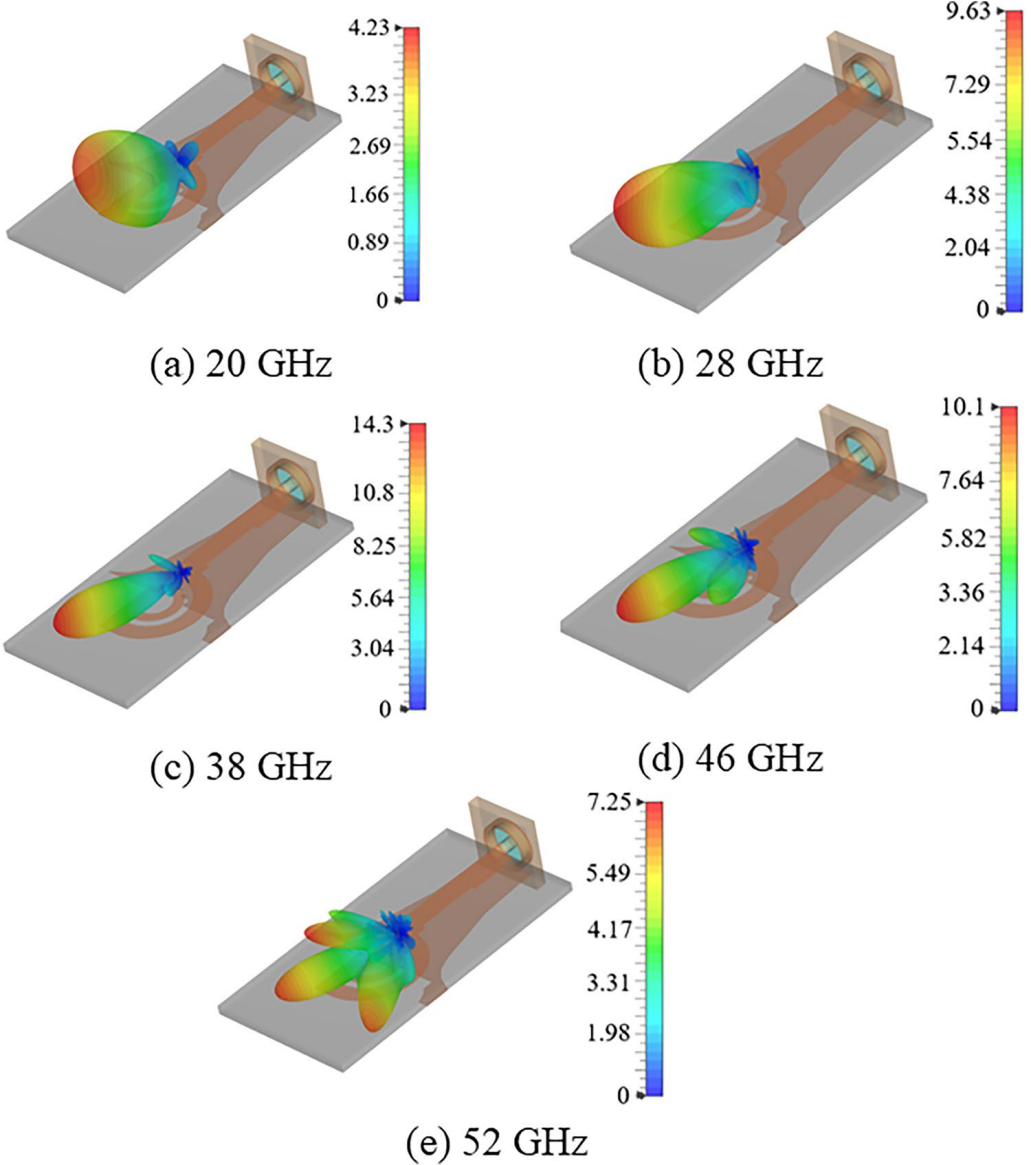
Simulated 3D linear realized gain plots of the double semi-circular rings antenna with grooves in top patch at (**a**) 20 GHz; (**b**) 28 GHz; (**c**) 40 GHz; (**d**) 46 GHz; and (**e**) 52 GHz.

Figure 12 compares the simulated reflection coefficients for the proposed antenna with and without grooves. It shows that the presence of two grooves in the feedline maintains the reflection coefficient value better than -10 dB over a frequency range from 11.5 to 65 GHz, closely matching the performance of the proposed antenna without grooves. Figure 13 illustrates the simulated realized linear gain plot for frequencies of 18 GHz, 28 GHz, 38 GHz, and 46 GHz. The antenna achieves a peak gain of 11.6 dBi at 40 GHz, as depicted in Fig. 11.

Figure 13 displays the simulated 3D linear realised gain plots at 18, 28, 38, and 46 GHz. It illustrates that the main lobe direction remains stable at $\textrm{65}^{\circ }$ ± $\textrm{10}^{\circ }$ across the operating bandwidth from 18 to 46.5 GHz. Although the proposed antenna offers a -10 dB IBW from 10 to 63.5 GHz, this method effectively controls the radiation beam within the range of 24 to 48 GHz. Below 24 GHz and beyond 48 GHz, the antenna transitions to other modes or resonant state, causing it to radiate the beam purely in the broadside direction, accompanied by increased SLLs. It is shown in Fig. 13a, e.

## Fabrication and experimental results

**Fig. 14:**
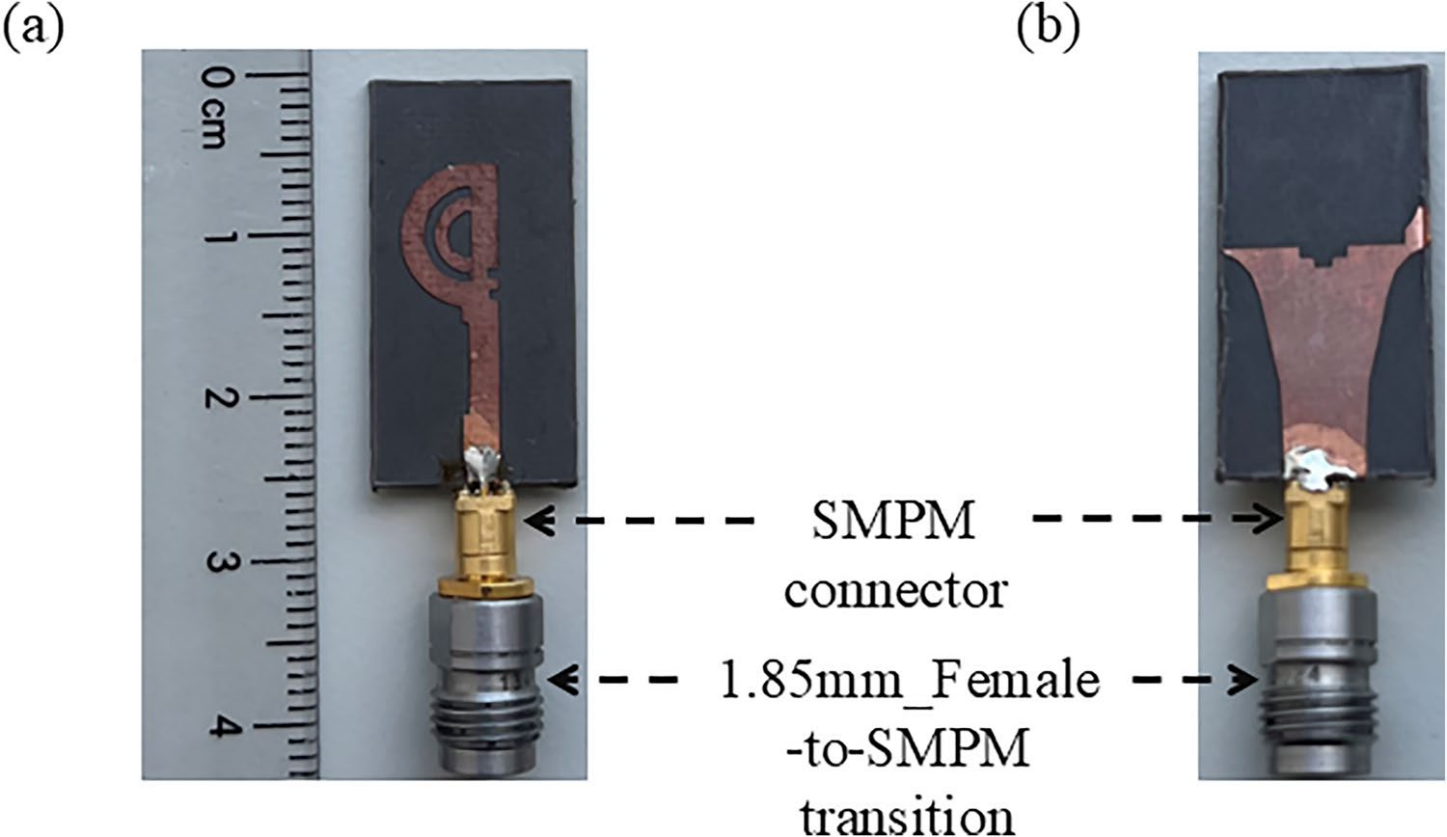
Fabricated prototype model of the double semi-circular rings antenna with grooves (**a**) top view; and (**b**) back view.

For analyzing the performance of the proposed design, a prototype model was fabricated, as depicted in Fig. 14. The overall dimensions are the same as those shown in Fig. 4c. The proposed antenna is fed by a 50 $\Omega$ 1.85 mm SMPM connector, suitable for frequencies up to suitable for frequencies up to 65 GHz. To measure the reflection coefficient of the proposed antenna, a ZVA vector network analyzer (VNA) from Rohde & Schwarz was utilized, covering a frequency range from 10 MHz to 67 GHz. This VNA features a 1.85 mm male connector at the end of the port, necessitating the use of a 1.85 mm female to SMPM transition to feed the signal into the proposed antenna. The insertion loss of this transition is negligible at lower frequencies and approximately 1.5 dB at higher frequency ranges. Hence, we have de-embedded the loss of connector and transition in all the measured results for fair comparison.

**Fig. 15:**
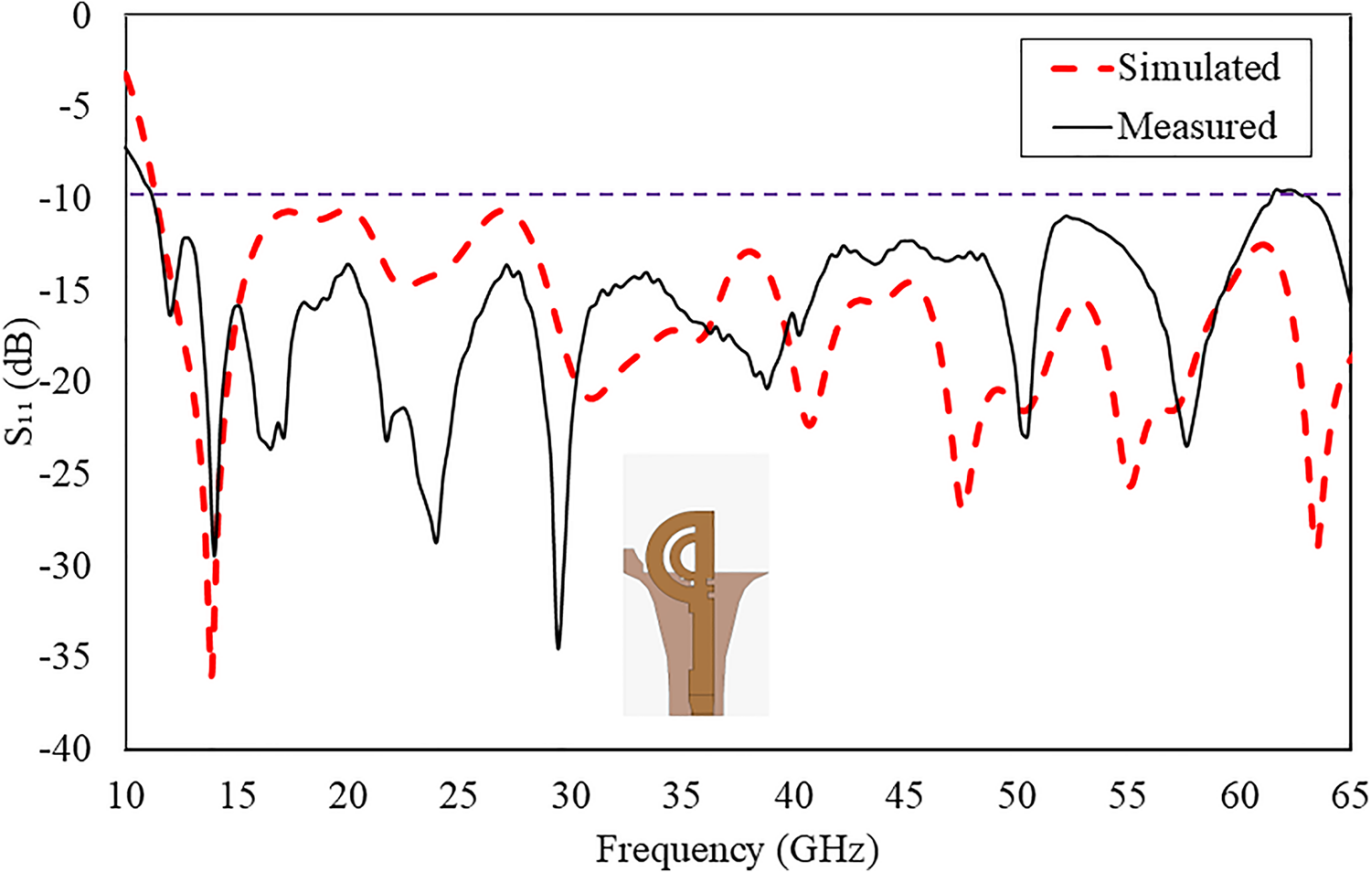
Comparison of measured and simulated reflection coefficients of the double semi-circular rings antenna with grooves.

Figure 15 illustrates the comparison between simulated and measured reflection coefficients of the proposed antenna. The measured results indicate that the proposed antenna supports a wideband impedance bandwidth of 40 GHz (approximately from 11.5 to 62.5 GHz, approximately 120%).

**Fig. 16:**
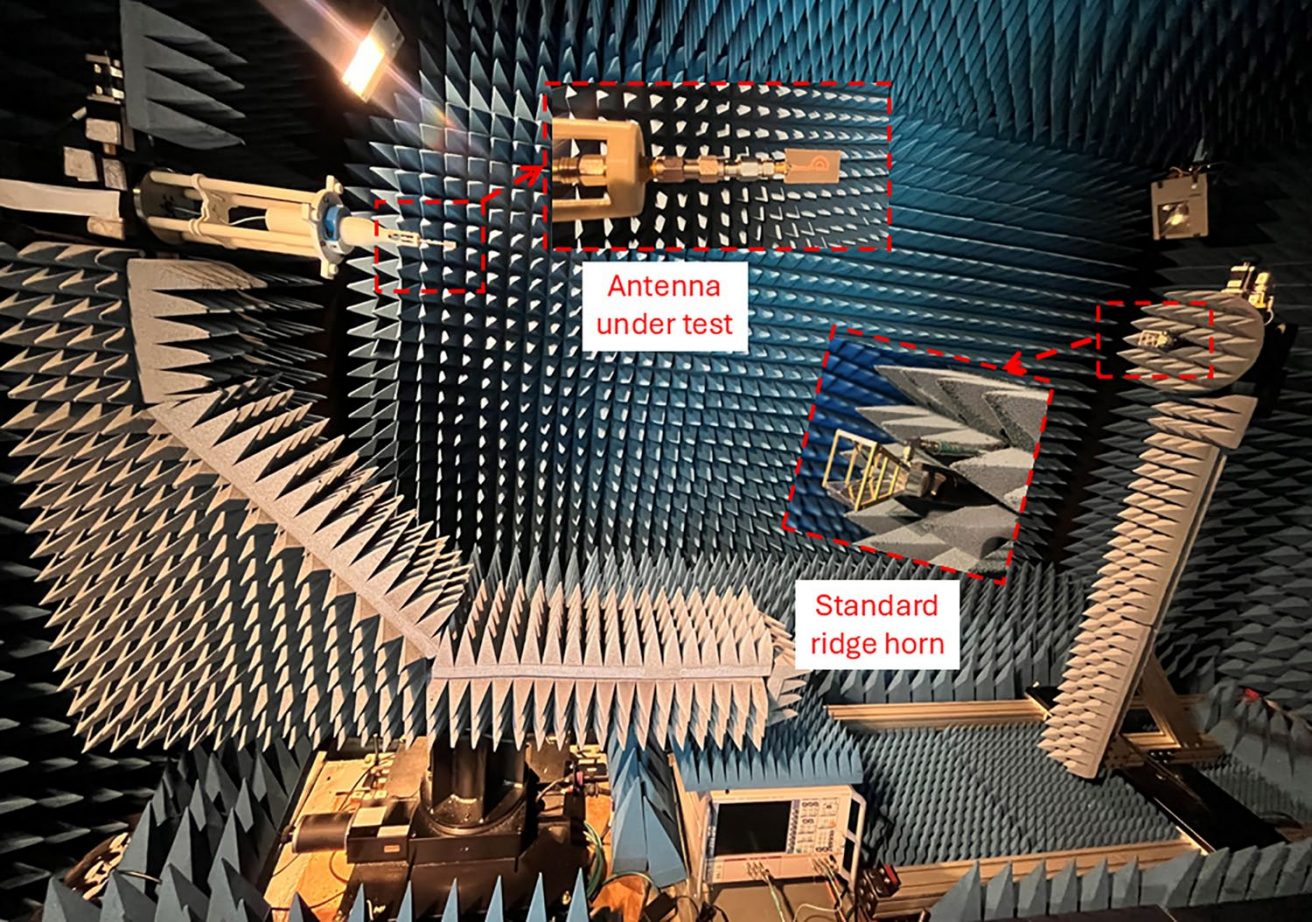
Test set-up for radiation pattern measurement in full anechoic chamber for the proposed antenna.

**Fig. 17:**
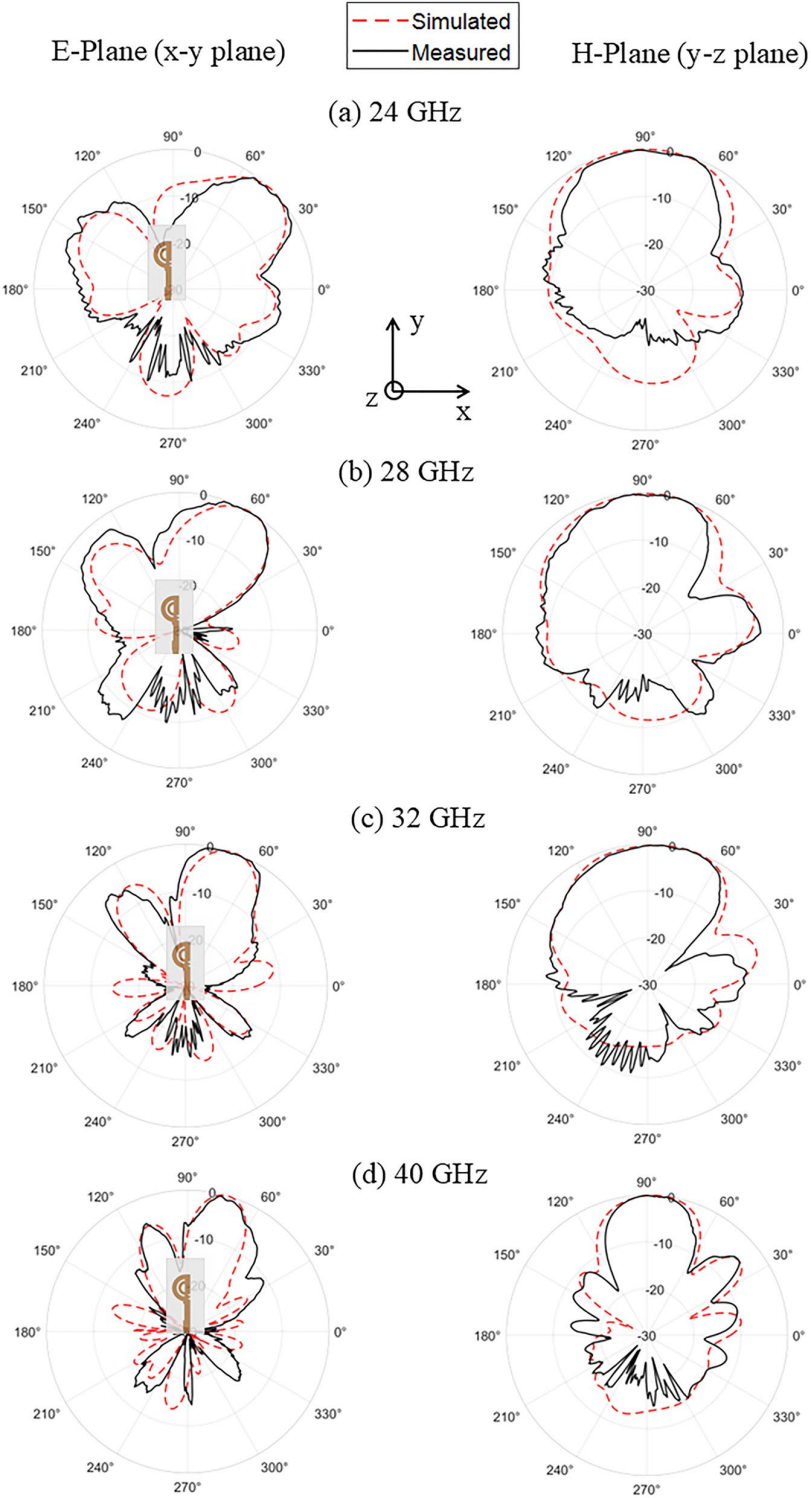
Comparison of simulated and measured normalized gain patterns in the E-plane and H-plane for the proposed antenna at (**a**) 24 GHz; (**b**) 28 GHz; (**c**) 32 GHz; and (**d**) 40 GHz.

The radiation pattern characteristics of the double semi-circular rings antenna were measured using the full anechoic chamber facility at Loughborough University. The test setup for radiation pattern measurement is depicted in Fig. 16. The 2D simulated and measured radiation patterns at 24 GHz, 28 GHz, 32 GHz, and 40 GHz are presented in Fig. 17.

**Fig. 18:**
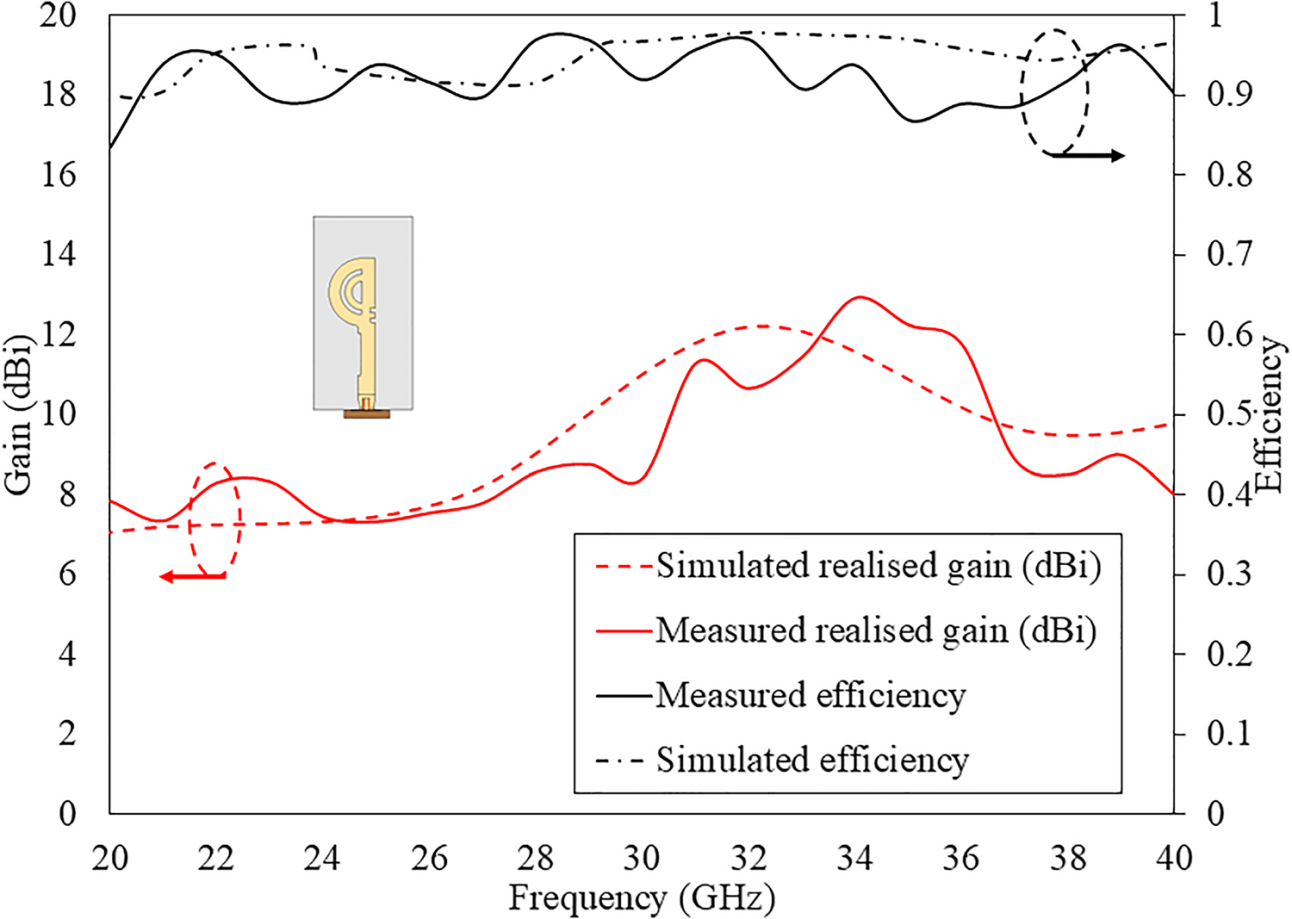
Comparison of measured and simulated gain, and efficiency plots for the proposed antenna.

Figure 18 illustrates the comparison between measured and simulated results of gain and efficiency over the frequency range of the proposed antenna. Both the gain and efficiency exhibit similar performance over the 10 dB IBW from 20 to 40 GHz (due to 40 GHz test facility available). The measured gain performance varies from 6.5 to 11.6 dBi over the operating frequency range.

## Comparison and discussion

The comparison of the double semi-circular rings with grooves as a tilted beam end-fire antenna with other reported works in terms of design technique, geometry of an antenna, dimension of the structure, bandwidth, gain, and efficiency is presented in Table 2. The proposed antenna has the smallest geometry and the widest operating bandwidth compared to previously reported tilted beam end-fire antennas^10,22,31–34^. Moreover, the proposed antenna can maintain the tilted beam direction almost constant over a frequency range from 24 to 48 GHz.

Most of the reported works used metamaterials or parasitic elements to tilt the beam or enhance the gain^22,32,33,35^. Incorporation of those elements may make the design more difficult to integrate into a communication system. The proposed design of antenna achieves a peak gain of 11.6 dBi without any metamaterials elements. Additionally, it has a wide operating bandwidth and achieves more than 84% radiation efficiency across the whole frequency band.

From the comparison shown in Table 2, it is evident that the proposed antenna offers several notable features, including a simple and planar geometry, wide operating bandwidth with a stable radiation pattern, high gain, and high efficiency. These characteristics position the proposed design as a promising candidate for future 5G mmWave applications.

**Table 2:** Comparison with other state-of-the art end-fire antennas. $*$ TB: Tilted beam.

Ref.	Technique	Size ($\lambda _0^3$)	TB’s Frequency Range (GHz) and FBW	Radiation Beam Angle	Peak Gain (dBi)	Radiation Efficiency (%)	SLL(dB)
^10^	Cavity backed slot array	1.6 $\times$ 1.1 $\times$ 0.02	27–28.6 (5.8%)	$\textrm{33}^\circ$	10.7	–	<-13.5
^22^	Yagi & patch deflector	1.67 $\times$ 0.83 $\times$ 0.02	4.8–5.2 (8%)	$\textrm{36}^\circ$–$\textrm{56}^\circ$	8.9	–	<-12.5
^23^	Bow-tie & artificial die. med	1.33 $\times$ 0.84 $\times$ 0.52	2.6–3.8 (5.7%)	$\textrm{35}^\circ$	10.4	–	< -8
^31^	Multilayer and array	2.15 $\times$ 0.2 $\times$ 0.25	23.5–30.5 (26.9%)	$\sim$ $\textrm{45}^\circ$	10.5	–	< -7
^32^	Patch array	4.2 $\times$ 1.87 $\times$ 0.02	23.4–33.9 (37.5%)	$\textrm{0}^\circ$	10.7	>90 (sim)	< -9
^33^	Single layer with FSS	2.39 $\times$ 1.54 $\times$ 0.04	25.9–32.0 (21.5%)	$\textrm{56}^\circ$–$\textrm{67}^\circ$	9.6	–	< -10
^34^	Vivaldi & FSS	2.29 $\times$ 2 $\times$ 0.04	26–29 (10.9%)	$\textrm{30}^\circ$–$\textrm{38}^\circ$	9	–	< -4
^35^	Asymmetrical bowtie	1.14 $\times$ 0.97 $\times$ 0.02	3.8–5.7 (32%)	$\textrm{30}^\circ$–$\textrm{45}^\circ$	5	–	< -8
^36^	Patch array	0.84 $\times$ 1 $\times$ 0.02	26–37.9 (37.2%)	$\textrm{90}^\circ$	10	>82	< -8
**This work**	**Double semi-circular rings**	**1.28** $\times$ **1** $\times$ **0.08**	**24–48 (75%) tilted beam**	**65**$\vphantom{0}^\circ$ ± **10**$\vphantom{0}^\circ$	**11.6**	>**84**	< -7

## Conclusion

This work presents a novel tilted-beam end-fire antenna based on a double semi-circular ring configuration for future microwave and millimeter-wave applications. Parametric optimization of the double-ring patch and ground plane demonstrates their strong influence on impedance matching and radiation characteristics. The proposed antenna achieves an ultra-wide impedance bandwidth from 11.5 to 62.5 GHz, enabling coverage of multiple microwave and mmWave frequency bands, including the 28 GHz, 38 GHz, and 60 GHz mmWave bands.

Importantly, while ultra-wideband impedance matching is achieved, the principal contribution of this work lies in the realization of a stable tilted-beam radiation pattern over a substantial portion of the operating spectrum. Measured results confirm consistent tilted end-fire radiation from 24 to 48 GHz (with measurements conducted up to 40 GHz), demonstrating the antenna’s suitability for directional mmWave applications. Within this band, the antenna provides a peak gain of 11.6 dBi and maintains a gain exceeding 6.5 dBi, highlighting robust radiation performance. The close agreement between simulated and measured results, particularly in terms of radiation patterns, gain, and efficiency, validates the proposed design approach. Future enhancements, including the integration of a 3D-printed dielectric lens or extension to array configurations, are expected to further improve the gain and beam control capabilities. Overall, the proposed antenna offers an effective solution for compact, high-gain, tilted-beam mmWave systems, with the stable tilted radiation performance constituting the key achievement of this study.

## Data Availability

Data is provided within the manuscript.
